# Temporal Modeling of Cognitive Decline: Health Patterns and Disease Progression in the All of Us Dataset

**DOI:** 10.1002/alz70861_108259

**Published:** 2025-12-23

**Authors:** Zahra Rahemi, Mohadeseh Hashemi, Meisam Omidi

**Affiliations:** ^1^ Clemson University, Greenville, SC USA; ^2^ Axle Informatics, La Marque, TX USA; ^3^ Marquette University, Milwaukee, WI USA

## Abstract

**Background:**

Timely identification of cognitive decline is critical for effective management of Alzheimer’s disease and related dementias (ADRD). Early insights into disease progression can inform preventive strategies and personalized care. This study leverages longitudinal data from the All of Us Research Program to examine comorbidity patterns and progression timing from mild cognitive impairment (MCI) to dementia.

**Methods:**

Using a concept‐name–based SQL pipeline in the All of Us Curated Data Repository (CDRv8), we identified participants with MCI and subsequent dementia diagnoses, classifying cognitive stages and calculating MCI‐to‐dementia intervals. Diagnosis timelines were aligned with comorbidity data to examine chronic condition distributions before, during, and after the transition. We applied dynamic time warping (DTW)‐based hierarchical clustering to comorbidity sequences for pattern discovery. Analyses focused on hypertension, type 2 diabetes, depression, and cardiovascular disease. Cluster validity was assessed using silhouette scores and the Davies–Bouldin index. Associations between comorbidities and conversion timing were evaluated using nonparametric tests.

**Results:**

Among 2,011 MCI participants, 281 progressed to dementia. The median time to conversion was 1,132 days (IQR: 594–1,765). Hypertension, diabetes, and depression were the most prevalent comorbidities and were each significantly associated with faster progression (p < 0.01). DTW‐based clustering revealed three subgroups: (1) rapid converters with dense cardiometabolic burden near diagnosis, (2) gradual converters with steady comorbidity accumulation, and (3) low‐burden profiles. Cluster separation was supported by validation metrics. Findings suggest that abrupt convergence of chronic conditions may signal accelerated transition to dementia.

**Conclusion:**

This study demonstrates the feasibility of deriving cognitive trajectories from All of Us data and reveals comorbidity profiles linked to progression speed. Findings support the development of explainable, fairness‐aware AI models to improve early risk prediction. Future work will integrate these models into clinical workflows to support timely, targeted dementia care, particularly for groups historically affected by diagnostic delays and limited access.

